# Preventive circadian medicine: improving health with sleep checkups

**DOI:** 10.1038/s44323-025-00047-z

**Published:** 2025-09-04

**Authors:** Yoichi Minami, Akifumi Kishi, Hiroki R. Ueda

**Affiliations:** 1 Department of Systems Pharmacology, Graduate School of Medicine, The University of Tokyo, Bunkyo-ku, Tokyo, Japan; 2 Laboratory for Synthetic Biology, RIKEN Center for Biosystems Dynamics Research (BDR), Suita, Osaka, Japan; 3 Directly under the President, Kurume University, Fukuoka, Japan

**Keywords:** Diseases, Circadian rhythms and sleep, Sleep

## Abstract

Sleep plays a crucial role in health, and illnesses can impact sleep. In this Perspective, we introduce the concept of “sleep checkups,” which use wearable devices to objectively and continuously measure sleep, providing feedback to enhance health and detect early signs of illness. Sleep checkups not only benefit individuals but also advance scientific understanding of sleep’s role in health, offering significant potential for participants and global public health.

## Introduction

Sleep has a significant impact on health^1^. Aspects of sleep, including sleep duration, insomnia, regularity, and chronotypes (morningness and eveningness) are associated with increased risk of cardiovascular disease, obesity, type 2 diabetes, and depression^2–4^. The American Heart Association counts getting healthy sleep (adequate sleep duration) as one of “Life’s Essential 8,” the key measures for improving cardiovascular health^5^. Sleep disorders are common in neuropsychiatric disorders such as major depression and bipolar disorder, post traumatic syndrome disorders (PTSD), schizophrenia, and Parkinson’s disease^6–8^. Blackwelder et al. conducted cross-sectional study using 2018 Behavioral Risk Factor Surveillance System (BRFSS) including 273,695 US adults (18–64) and found 13% of participants experienced inadequate sleep (less than 6-hrs sleep) and these people were about 2.5 times more likely to have frequent mental distress (self-reporting 14 days of mental health status as “not good”)^9^. Furthermore, sleep duration has been reported to be associated with all-cause mortality^10,11^. Both short and long sleep increase the risk of mortality. Also, irregular sleep increases the risk of mortality^12,13^. Our global society is supported by shift workers, and shift work causes health problems, at least some of which arise from circadian misalignment^14^. Wittmann et al. notes that the mental and physical effects of the repetition of differences in activity time between socially constrained weekdays and socially unconstrained free days (holidays) are like jet lag, which they call social jet lag^15^. The effects of sleep and circadian rhythm misalignment on the body and mind are being underestimated, and countermeasures are needed^16^. Recently, in addition to sleep medicine, the concept of sleep health, which takes a multi-dimensional approach to sleep and health issues, has become widespread^17^. Ko et al. pointed out that consumer sleep technologies are becoming popular among the general public for purposes such as sleep improvement and monitoring. They illustrated this by noting that Fitbit and Jawbone are top-selling consumer health products, and that the highest-funded health device on Kickstarter was a sleep monitor^18^.

Not only those that record acceleration alone, but also those that can simultaneously record other modalities (e.g., heart rate and body temperature) are available and are rapidly becoming popular around the world as a tool for accurate sleep measurement^19^. Accelerometer-derived sleep data collected by wearables is considered a promising digital biomarker for mood disorders^20,21^. Many trials are underway to detect sleep disorders with wearables to establish an objective and convenient system for diagnosing sleep disorders^22^. We propose incorporating routine sleep measurement into health checkups to maintain and improve overall health, monitor for early signs of diseases, and detect pre-symptomatic conditions—what we term as “sleep checkups.” Sleep can be quantitatively evaluated, and using wearables allows for continuous measurement over multiple days in daily life, resulting in stable data that reflects normal living conditions. Participants can achieve health improvements through objective data-based sleep management and sleep hygiene.

**Fig. 1:**
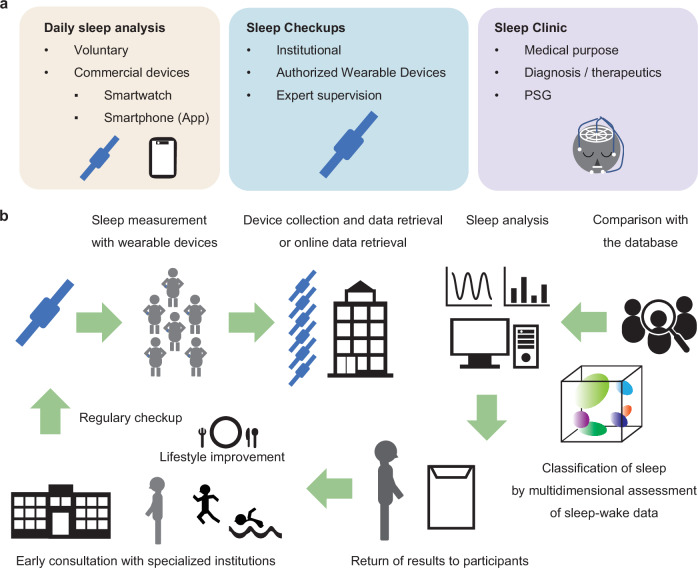
Sleep checkups represent a preventive medical approach aimed at promoting participants’ health and well-being. **a** Sleep checkups bridge the gap between voluntary self-monitoring using commercial products and the diagnostic and therapeutic processes in sleep clinics. **b** Flow of sleep checkups. Participants wear devices (wearables) to measure their daily acceleration for at least a week. (Top left) The devices are returned to the analysis center for data collection, or alternatively, an online data collection system is utilized. (Top middle, right) The analysis center analyses participant data and selects subjects for attention. (Bottom) Data is returned to participants and used to improve their lifestyle. If necessary, participants are advised to undergo specialized examinations at advanced medical institutions, supporting the early detection and treatment of diseases. These processes are repeated periodically (e.g., annually) as health checkups.

In this article, we propose the concept of “sleep checkups” to promote and maintain health through regular sleep measurement. First, we introduce the concept of “sleep health,” followed by a description of quantitative analysis using wearables. Next, we provide a detailed explanation of the sleep checkups (Fig. 1a). We envision sleep checkups not as clinical services, but as a system integrated into social infrastructure. Technologically, sleep checkups involve quantitative sleep measurements and analyses using wearable devices, including sleep pattern classification, with the results used to provide tailored feedback to users. These services are expected to be delivered by qualified professional organizations, either for-profit or nonprofit. Sleep checkups aim to improve sleep and promote health, identifying individuals who need advanced medical care (Fig. 1b). Finally, we discuss the proof of concept using large-scale data and the scientific significance that sleep checkups will offer.

## Approaches to sleep issues: sleep medicine and sleep health

### Sleep medicine focuses on sleep disorders

Sleep medicine focuses on sleep disorders, confirms symptoms, and advances treatment. The International Classification of Diseases 11th Revision (ICD-11) was adopted by the World Health Assembly in 2019 and became officially in effect from 2022^23,24^. The ICD-11 includes a chapter on sleep-wake disorders, like insomnia disorders, hypersomnolence disorders, sleep-related breathing disorders, circadian rhythm sleep-wake disorders, sleep-related movement disorders, and parasomnia disorders^25^. Insomnia is the most common sleep disorder in the adult populations. Although prevalence rates vary from 5% to 50%, likely due to differences in definitions (symptoms, disorders, dissatisfaction), population-based data generally indicate that about one-third of adults (30–36%) experience at least one nocturnal insomnia symptom^26^. Morin et al. conducted a 5-year follow-up study of 3,703 individuals (aged 18–95) and reported that 13.9% of subjects who had relatively good sleep at the baseline period developed insomnia symptoms^27^. The prevalence of narcolepsy in the general population is approximately 44.3 per 100,000 persons, and delayed sleep phase syndrome (delayed sleep-wake phase disorder), common in adolescents, is 3.3%^28,29^. Older adults tend to have sleep problems and have a high incidence (20–40%) of sleep-disordered breathing (SDB)^30^.

### Sleep health explores various issues related to sleep from multiple perspectives

Sleep health goes beyond diagnosing and addressing sleep disorders to examine a wide range of issues related to sleep and the relationship between sleep and physical and mental health from biological, public health to sociological perspectives. Dr. Buysse defines sleep health as follows:

*“Sleep health is a multidimensional pattern of sleep-wakefulness, adapted to individual, social, and environmental demands, that promotes physical and mental well-being. Good sleep health is characterized by subjective satisfaction, appropriate timing, adequate duration, high efficiency, and sustained alertness during waking hours*”^17^.

He emphasized the importance of developing multidimensional measures for sleep health and proposed the SATED scale (**S**atisfaction, **A**lertness, **T**iming, **E**fficiency, **D**uration), or Ru-SATED scale, which includes **R**egularity^17^. A multidimensional assessment derived from Ru-SATED is being discussed in pediatric medicine, and Meltzer has proposed B-SATED, which adds **B**ehaviour in place of Regularity^31^. Although the original Ru-SATED is a questionnaire, Wallace et al. showed that this concept can be applied to objective actigraphy data^32^. Factorial analysis revealed that the 28 sleep-related indices (such as sleep-wake rhythm amplitude, sleep duration, and wake/sleep times) can be grouped into five factors. Based on their content, these factors are believed to represent Timing, Efficiency, Duration, Alertness, and Regularity^32^. The National Sleep Foundation (NSF) developed multidimensional sleep health analysis indices named Sleep Health Index (SHI) and Sleep Satisfaction Tools (SST), which assess the general population’s sleep satisfaction^33,34^. The NSF and other authorized organizations reported consensus papers for not only sleep duration, but also sleep quality and sleep regularity^35–38^.

## Quantitative analysis and detection of sleep-wake abnormalities

### Wearables are suitable for long-term quantitative sleep monitoring and large-scale measurements

Common methods of sleep measurement include sleep diary methods, polysomnography (PSG) tests, and wearable-based measurements. Sleep diaries are advantageous as they are easy to use and provide a subjective assessment, but accuracy and the effort required can be an issue. PSG is a technique that assesses sleep status and detects sleep disorders by measuring various physiological signals throughout the night, such as the electroencephalogram (EEG), electromyogram (EMG), electrooculogram (EOG), electrocardiogram (ECG), respiratory parameters, and pulse oximetry^39^. PSG is the gold standard for measuring human sleep, but it is not suitable for large-scale continuous day-to-day measurement analysis. Wearables automatically and objectively record sleep-wake activity. The devices are suitable for mass production and relatively inexpensive compared to specialized medical devices such as PSG. Wrist-worn actigraphy is the most common, but other types of wearables are also available, such as ring-shaped devices and simplified EEG headbands. In the white paper summarizing the 2018 World Sleep Workshop, Depner et al. extended the concept of “wearables” to “nearables” that collect data close to the user’s environment, as well as sensors that are detected inside the user’s body, such as ingestible deep body temperature measurement devices (“ingestible”)^19,40^.

### The low specificity of wearable devices has been pointed out

The effectiveness of sleep-wake classification is algorithm-dependent and in many cases, has high sensitivity (detecting true sleep) and high accuracy (detecting true sleep and wake periods), but not high enough specificity for distinguishing true wake periods^40^. Epoch-base comparison of classifications by PSG as ground truth, specificity from the report by Kosmadopoulos et al. was 26.9% (Actiwatch-64), Markwald et al. was 37.0% (Actiwatch64), Walch et al*.* was 54.1% (Apple Watch), and de Zambotti et al. were 42.4% (Fitbit) and 48% (Oura Ring)^41–45^. Chinoy et al. compared seven commercially available sleep trackers and found that, although there were differences among devices, in general, the sensitivity of sleep detection was comparable to the PSG (93%). However, it was not effective in detecting wake, with specificity ranging from low to moderate (18–54%)^46^. Lujan et al. noted that this is because both the devices and algorithms are optimized to measure sleep at night. They also noted that while these devices are sensitive and relatively accurate, their low specificity requires caution in interpreting sleep duration^47^.

**Fig. 2:**
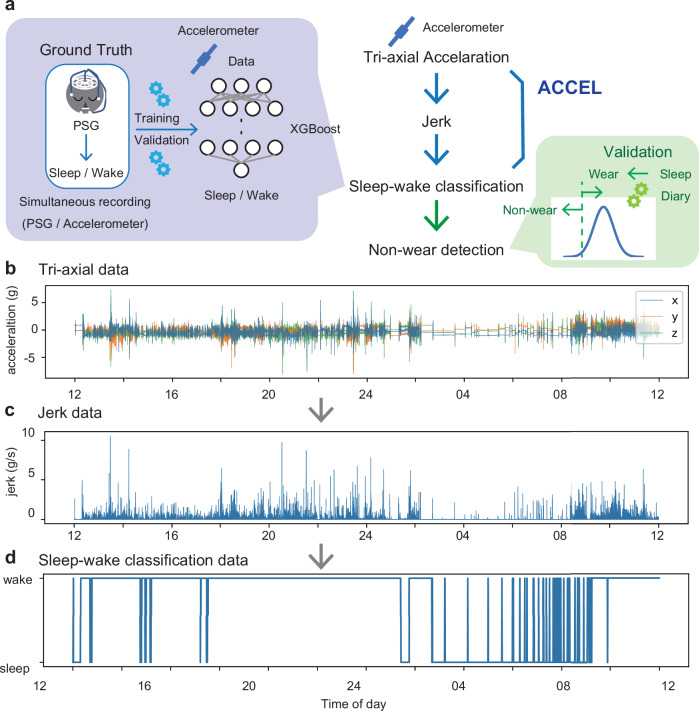
The sleep–wake detection algorithm (ACCEL) determines sleep and wake states by utilizing the derivative values of acceleration. **a** ACCEL, like other algorithms, classifies wakefulness and sleep based on triaxial acceleration data. Uniquely, the algorithm uses the jerk calculated from acceleration. The sensitivity, accuracy, and specificity of the sleep-wake classification algorithm are increased by using machine learning with PSG data as the ground truth (purple). It is possible to determine non-wear by considering continuous immobility time (green). Representative examples of tri-axial acceleration data (**b**), its time derivative jerk data (**c**), and classified sleep-wake data (**d**). High activity is classified as wake, and low activity as sleep. Short awakenings during sleep are also detected (**d**).

We have developed a sleep-wake classification algorithm called ACceleration-based Classification and Estimation of Long-term sleep-wake cycles (ACCEL)^48^ (Fig. 2a). ACCEL is an algorithm developed to enhance the accuracy, sensitivity, and specificity of sleep-wake classification using machine learning (XGBoost) (Fig. 2a). It utilizes the derivative of triaxial acceleration (jerk) to reduce individual variability without relying on device-specific functions (Fig. 2b–d). We reported that ACCEL achieved high accuracy (91.7%), high sensitivity (96.2%), and high specificity (80.1%) for data measured with our original device (SONY)^48^. Katori et al. demonstrated that the ACCEL algorithm can be applied to real-world data measured using different devices (AX3, Axivity). In this study, the ACCEL method achieved high accuracy (93.2%), high sensitivity (97.2%), and high specificity (82.2%)^49^. Wake during night-time sleep affects satisfaction with sleep, and high specificity in sleep-wake classification by wearables will contribute to the evaluation of sleep efficiency and sleep quality.

## Periodic sleep checkups for health maintenance and improvement

### Sleep checkups: Between personal sleep monitoring and sleep medicine

De Zambotti et al. summarized the current state of wearable sleep technology, highlighting the use of traditional actigraphy, consumer-grade multi-sensor devices, and clinical-grade trackers across self-monitoring, research, and clinical applications. They noted the increasingly blurred lines between consumer and clinical devices, as well as between wellness and medical tools^50^.

From both research and clinical perspectives, actigraphy serves as a valuable tool for investigating sleep, neurodegenerative disorders (e.g., Alzheimer’s and Parkinson’s diseases), and mental health conditions, including insomnia and depression. The UK Biobank is a large prospective study involving 500,000 people aged 40–69, recruited between 2006 and 2010. Seven sleep-related data fields (category 100057), covering sleep duration, dozing, napping, insomnia, sleepiness, snoring, and chronotype, as well as an activity monitor data set (category 1008 and its subcategories) capturing physical activity using wearables (AX3, Axivity) over seven days for approximately 100,000 participants, which can be used to quantify sleep^51^. Retrospective analyses using UK Biobank actigraphy data have shown that higher circadian rhythm amplitude is associated with reduced risks of various health outcomes, including cardiovascular, metabolic, respiratory, infectious diseases, cancer, and all-cause mortality^52^. Lyall et al. reported that reduced circadian amplitude was linked to increased risk of psychiatric disorders such as major depressive disorder and bipolar disorder, along with poorer subjective well-being (e.g., increased loneliness, lower happiness and health satisfaction, slower reaction times)^53^. Similarly, Bian et al. identified a U-shaped relationship between sleep duration and dementia incidence, with short sleep (<7 h) significantly increasing risk. Notably, the benefits of regular sleep patterns were observed only among those with short or long sleep durations^54^. Gubin et al. further advocate for the use of wearables from a “Circadian Health” perspective, emphasizing 24-hour behavioral rhythms beyond sleep alone^55^.

Previous studies have sought to bridge the gap between self-monitoring and clinical sleep assessment. For example, Pinilla et al. provided a comprehensive review of obstructive sleep apnea (OSA) screening methods, including full and simplified PSG, various wearable devices (wrist-, finger-, and body-worn), and nearables^56^. STOP/STOP-Bang questionnaire was developed as an easy-to-use screening tool for OSA^57^. Both the Epworth Sleepiness Scale (ESS) and the Pittsburgh Sleep Quality Index (PSQI) are widely used questionnaires that measure different aspects of sleepiness and sleep quality^58,59^. Recent efforts also include the development of machine learning-based screening tools for disorders like insomnia and OSA^60^. Positioned as an extension of these approaches, sleep checkups aim not at diagnosing specific diseases, but rather at providing systematic, objective feedback using wearable devices. As a form of primary prevention, they return evidence-based sleep-related information to participants, with the goal of promoting health awareness and behavioral change.

Sleep coaching encompasses sleep hygiene education and cognitive-behavioral techniques aimed at improving sleep quality^61^. Holzinger and Kloesch have introduced a holistic, non-pharmacological model based on Gestalt therapy for addressing non-restorative sleep^62^. Sleep coaching represents a gentle, individualized intervention that supports behavior change through daily sleep monitoring and personalized feedback via apps or sleep counselors. Our proposed “sleep checkups” shares conceptual similarities with “sleep coaching” but differs in its structure and purpose. It is designed as a community-based health maintenance framework, implemented by non-profit institutions or authorized for-profit organizations such as clinical laboratories. Whereas sleep coaching emphasizes behavioral intervention, sleep checkups focus more on raising awareness through regular assessment of sleep status, thereby inducing behavioral change. Like routine health checkups aimed at detecting latent health risks, sleep checkups enable individuals to recognize potential sleep-related risks and encourage improvements in sleep hygiene (Fig. 1a).

### Health checkups contribute to the early detection and treatment of diseases and maintain and promote good health

Health checkups are a system designed to assess one’s health status, identify early signs of disease, and manage health risks. Standard health checkups examine health status through several tests, such as blood tests, urinalysis, and vital sign evaluation for health management. Although its significance and improvements are being tested for the next generation, proper health checkups can lead to early detection of illness among participants and allow healthcare providers to devote more specialized resources to patients in need^63^. In Japan, regular health checkups are the norm and have been a cornerstone of public health policy, serving as a key opportunity for early detection of diseases and health promotion. Ikeda et al. point to the health consciousness among Japanese as one of the reasons “what made the Japanese healthy,” and highlight the existence of regular health checkup systems and a systematic checkup of the whole body commonly referred to as “human dry dock”^64^.

### Sleep checkups aim to improve sleep, promote health, and contribute to preventive circadian medicine through sleep measurement

Honaker first used “the Sleep Checkup” as a service delivered by behavioral health providers in two urban primary care clinics^65^. This “Sleep Checkup” is a practical tool to support pediatric care —a brief assessment of sleep patterns and concerns, followed by tailored advice, educational materials, and, when necessary, referral to a sleep specialist, considering the diverse sleep needs and family contexts^66^. On the other hand, our concept of “sleep checkups” is modeled after general health checkups (see “Health checkups contribute to the early detection and treatment of diseases and maintain and promote good health”). It is positioned as a primary prevention strategy aimed at promoting personal health and enabling early detection of potential disorders through periodic population-based assessments (e.g., annually) (Fig. 1a).

The proposed framework for sleep checkups consists of four key steps: (1) Participants wear a device for a designated period (e.g., at least one week, see below) to measure sleep during daily life; (2) Data are returned to the provider, either via device return or remote upload; (3) Sleep phenotypes are analyzed using multidimensional metrics; and (4) Participants receive personalized feedback. Through regular participation (e.g., annually), individuals can monitor changes in sleep and overall health, gain insight into their sleep patterns, and improve sleep hygiene and lifestyle behaviors (Fig. 1b).

This model is already being implemented in practice, particularly within employee wellness programs, where employers subsidize the cost. In these cases, sleep tech companies provide research-grade wearable devices (e.g., AX3), pre-configured and ready-to-use. Participants wear the devices during daily life for one week, return them afterward, and the data are processed and analyzed by the provider. Daily sleep diaries and short health surveys are also completed by participants and incorporated into the analysis. Personalized reports include visualizations of sleep patterns during the measurement period, and key metrics such as total sleep time, number of nocturnal awakenings, and sleep regularity. These are compared with reference values to help participants understand their current sleep status. As in regular health checkups, reports provide tailored feedback: participants with potentially concerning patterns are advised to seek further evaluation from clinical specialists, while those without significant issues receive recommendations for optimizing sleep hygiene and maintaining healthy sleep habits.

Participation involves certain burdens: individuals must wear devices continuously for several days, complete daily logs, and manage device receipt and return. The American Medical Association CPT® code 95803 requires a minimum of 72 hours for billing actigraphy as a stand-alone service. However, clinically, 7–14 days of recording is recommended to capture both weekday and weekend sleep patterns, with 14 days preferred for individuals with irregular schedules^67^. At present, a 7-day protocol is adopted, balancing device turnaround efficiency, the ability to include both weekdays and weekends, and prior success in estimating sleep patterns from 7-day actigraphy in large-scale studies^49,51–53^. Providers, in turn, must invest in sufficient numbers of validated devices, as well as research and development for data analysis and interpretation. Operational costs include personnel for device setup, data retrieval, analysis, and logistics. To ensure quality, supervision by sleep specialists and/or sleep scientists is required. As this system operates outside the domain of routine clinical care, it does not increase the burden on existing healthcare services. For non-working populations such as children or older adults, financial barriers remain; thus, we propose a publicly funded sleep checkup program modeled on existing national health screening systems.

### General concerns about health checkups and sleep checkups

It is important to note that there are opinions suggesting the need for evaluations of the effectiveness of health checkups. Liss et al. reported that general health checks (health checkups) did not demonstrate a reduction in mortality or cardiovascular events; but, they were associated with increased recognition and treatment of chronic diseases, improved control of risk factors, and some other outcomes^68^. The Organization for Economic Co-operation and Development (OECD) recommends that, in addition to evidence on the effectiveness of health checkups, economic evaluation should also be promoted^69^. The specific health checkups (Tokutei Kenshin) in Japan aim to prevent and detect lifestyle-related diseases early and promote health maintenance and improvement through specific health guidance before costly treatments become necessary^69^. Similarly, sleep checkups hold the potential to prevent rising medical costs by enabling early treatment of sleep disorders. Concerns associated with traditional health checkups, such as radiation exposure and increased medical expenses^69^, are less applicable to sleep checkups using wearables. Wearables are non-invasive and pose no risk of side effects after measurement. While they are relatively inexpensive compared to specialized clinical tools such as PSG, they are still costly. For sleep assessments aimed at primary prevention, a “Bring Your Own Device” (BYOD) strategy using consumer-grade wearable devices that meet predefined validation criteria may be effective^70^. By exchanging data via the cloud, operational costs could be further reduced. While practical issues must be considered, the overall outlook remains optimistic. Capturing changes and assessing whether stable sleep is being achieved can also be beneficial. In that sense, establishing a system for daily measurements and receiving feedback in near real-time would be ideal. However, considering costs and operational methods, this might be excessive for a primary prevention framework. Instead, it would be more meaningful to create a system that allows for regular assessments, enabling individuals to make temporal comparisons and fostering stability in their sleep patterns.

Missing data is an inherent challenge in collecting data via wearable devices. Chapter 4 of the Society of Behavioral Sleep Medicine (SBSM) Guide to Actigraphy Monitoring addresses this issue and provides the following recommendations: (1) exclude periods at the beginning and end of recordings when the device was not worn, (2) identify and flag segments with missing data, and (3) document any abnormal or atypical movement patterns observed during monitoring^67^. Several methods have been proposed for addressing missing data. Multiple imputation assumes an underlying distribution for the missing values and generates multiple random draws to reflect imputation uncertainty. More recently, advances in artificial intelligence and machine learning have led to the development of deep learning–based approaches for imputation. Jang et al. proposed a deep learning model to impute missing actigraphy data and demonstrated its high performance^71^. Lee et al. introduced a neural network model called SOMNI, which can impute missing sleep data using either individual-level or group-level inputs^72^. These provide insights into potential solutions to enhance data reliability and accuracy.

## Sleep phenotyping and clustering: considerations for implementing sleep checkups

### Biobanks are important resources for experimentally testing laboratory findings with real-world data

**Fig. 3:**
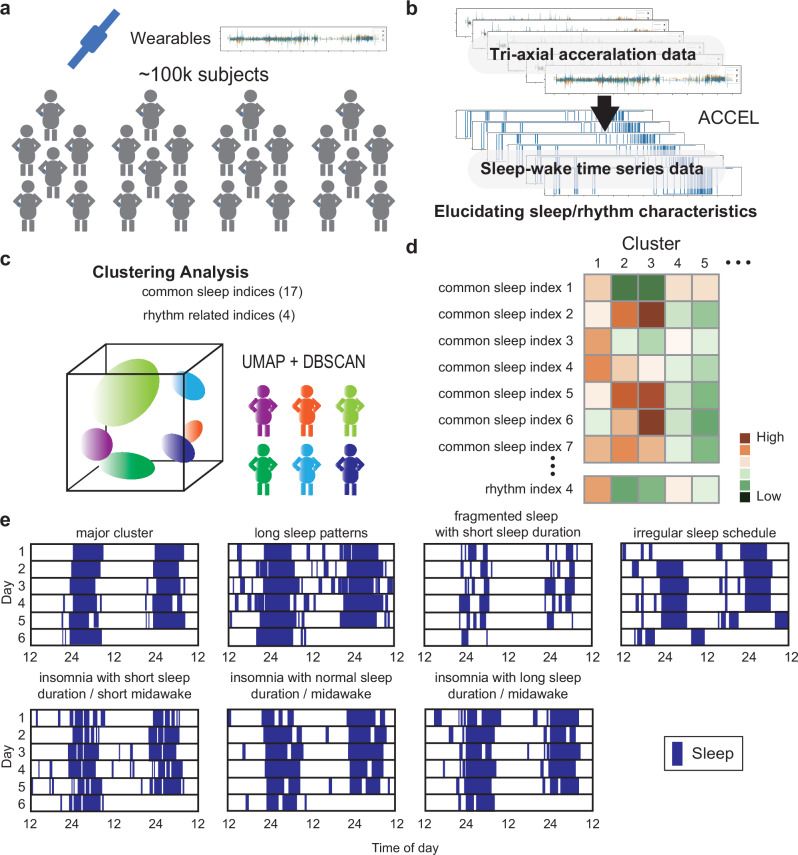
Utilizing large-scale time-series datasets and multivariate analysis allows for the comprehensive depiction of the sleep landscape. **a** Large datasets (the UK Biobank, approximately 100,000 participants) were used to identify general sleep patterns. **b** Tri-axial acceleration data were processed using ACCEL to generate sleep-wake time series, from which 17 common sleep indices and 4 rhythm-related indices were calculated. **c** Clustering analysis was performed using statistical methods (UMAP + DBSCAN). Color-coded clouds in the cube represent identified clusters. **d** Each cluster exhibits a different index pattern. **e** Representative sleep-wake patterns of individuals from the clusters. Blue boxes indicate sleep. Clusters identified include long sleepers (upper middle left), fragmented short sleepers (upper middle right), irregular sleep patterns (upper right), and clusters containing various types of insomnia.

In sleep checkups, it is essential to identify high-risk groups based on sleep phenotypes. To demonstrate the feasibility of this approach, we present two “sleep landscape” analyses that utilize large scale data for the multidimensional evaluation and grouping of sleep patterns. Katori et al. successfully identified 16 distinct sleep pattern clusters using accelerometer data from approximately 100,000 participants in the UK Biobank (Fig. 3). Sleep-wake classification was performed using ACCEL, from which we derived 21 indices—including 17 conventional sleep indices and 4 rhythm-related indices. For clustering, we applied uniform manifold approximation and projection (UMAP) for dimensionality reduction, followed by density-based spatial clustering of applications with noise (DBSCAN). Each resulting cluster represented a unique sleep pattern, including chronotypes such as “night owl” and “early bird” (Fig. 3a–d). Notably, some clusters exhibited insomnia-like phenotypes (Fig. 3e). One such cluster, characterized by fragmented sleep without prolonged rest periods, was labeled “short and fragmented sleep.” These findings highlight the potential of sleep measurement and phenotypic analysis to identify individuals who may benefit from targeted sleep health interventions^49^.

Viswanath et al. conducted a similar analysis using the smart-ring wearable device (Oura Ring), examining 4,682,978 (91.89%) out of 5,095,798 potential nights of data (33,152 individuals, age = 44.4 ± 12 years (mean ± std.)). This study emphasizes longitudinal changes in intra-individual variation over time, an aspect that has been missing in previous models. They investigated whether an individual’s sleep phenotype changes over time and if such changes offer new insights into participants’ health state (e.g., insomnia, influenza, fever, COVID-19). They identified 13 sleep phenotypes related to sleep quality, with individuals transitioning between these phenotypes over time. Importantly, patterns of these transitions provided more information about chronic and acute health conditions than static phenotypic membership alone. Although further research is needed to validate these findings across different conditions and populations, the results suggest that incorporating temporal sleep dynamics could enhance health screening tools^73^.

## What sleep checkups bring

### From sleep checkups to mental and brain health

Sleep checkups, which aim to assess health status through sleep, represent an attempt to utilize sleep as a digital biomarker for internal physiological conditions. One area where this approach could contribute is mental/brain health. There is a strong association between sleep and psychiatric disorders, and alterations detected through accelerometry have been linked to various mental health conditions, suggesting the potential for detecting psychiatric disorders through sleep monitoring^53,74^. Just as standard health checkups include vital sign assessments such as blood and urine tests, sleep checkups could also incorporate additional components—such as questionnaires on mental health status—to serve as a tool for mental health screening. Lim et al. reported that prospective observational cohort studies have demonstrated that combining sleep–wake data with individuals’ history of mood episodes enables effective prediction of future mood episodes and may enhance the management of mood disorders^75^. Furthermore, if sleep screening were to evolve into a system capable of real-time monitoring and feedback, it might be possible to detect arousal levels and drowsiness and provide interventions to help people spend the day in a healthy brain state. Two studies employing mathematical models of sleepiness and alertness demonstrate that personalized sleep interventions—based on sleep–wake patterns obtained from wearable devices and computational modeling of sleep pressure and circadian rhythms—can be highly effective. By gaining insight into individual patterns of sleepiness and alertness and adjusting sleep schedules in alignment with one’s circadian rhythm, even individuals with irregular routines, such as shift workers, can enhance alertness and reduce daytime sleepiness^76,77^.

### Contribute to sleep health through the accumulation of large-scale objective data

On behalf of the World Sleep Society Global Sleep Health Taskforce, Lim et al. pointed out that the importance of “sleep health” is underestimated in public health agendas and educational institutions in most countries worldwide. They recommend promoting sleep health through education (to enhance sleep and circadian health education and awareness), research (by collecting and centralizing standard sleep and circadian data in every country), and public health implementations (including sleep health in public health policies)^78^.

Other than public health implementations, the most significant contribution of sleep checkups to sleep health and public health will be the accumulation of large-scale, objective sleep data across diverse populations. Traditional large-scale public health surveys predominantly rely on questionnaires, which are globally insufficient^78^. With appropriate ethical considerations, handling sleep checkups data will enable the analysis of large-scale, quantitative data that has previously been lacking. Further analysis in combination with other health indicators of the individual can not only provide deeper insights into the relationship between health and sleep but also deepen the significance of the sleep checkups itself. This is expected to contribute to the future incorporation of sleep as a digital biomarker for disease diagnosis. Interest in sleep is high, and providing people with feedback on scientific findings could also contribute to improving sleep literacy.

In the future, as genomic information becomes utilized for precision medicine in health risk management, linking sleep information with genomic data is expected to advance the genetic understanding of sleep. At present, some biobanks store sleep-related data and whole-genome sequence data from the same participants. Examples are the Million Veteran Program (MVP), the Japan Multi-Institutional Collaborative Cohort (J-MICC), the National FINRISK study, the Trøndelag Health Study (The HUNT study), and the UK Biobank^79–82^. These datasets have made a significant contribution to the genetics of human sleep through genome-wide association studies (GWAS). following are UK Biobank examples: Li and Zhao conducted a GWAS and identified 53 loci associated with sleep, obesity, psychiatric, and neurological disorders^83^. Fei et al. identified 68 genes associated with sleep-related traits at exome-wide significance using data, including *VPS8*, *CNNM2* (insomnia symptoms), *RGS16* (Chronotype), *HCRTR2* (Ease of getting up in the morning), *PATJ1* (Daytime sleepiness), *CRHR1* (Daytime napping), *MSRB3* (Snoring), and *CGN* (sleep apnea)^84^. This amount of GWAS results will not only pave the way for elucidating the genetic background of sleep disorders but also provide clues for enhancing precision sleep health tailored to each individual.

### Understanding the diversity and generality of sleep from large-scale objective data

Sleep patterns vary widely depending on social and living environments. Sleep phenotypes also differ between individuals, even in the same environment and, moreover, even within the same person from day to day. Large-scale studies contribute to understanding the diversity and generality of human sleep. As examples, Ong et al. analyzed the sleep of 553,559 nights from 23,680 users aged 15–80 years in Oceania and East Asia, as captured by Fitbit devices, and found that East Asians had later bedtimes and they reported that the sleep duration was shorter^85^. Jonasdottir et al. used Sony’s SmartBand to analyze the sleep of 69,650 adult non-shift workers aged 19–67 in 47 countries. The study confirmed an age-related decrease in sleep duration and earlier onset and offset of sleep and revealed differences between weekdays and weekends^86^. Using a similar smart band, Minor et al. analyzed the sleep of 47,628 participants from around the world, showing how rising temperatures lead to shorter sleep times^87^. Kuula et al. analyzed the sleep and sleep duration of 17,355 Polar wrist-worn sleep tracker users aged 16–30 years in 107 countries, primarily in Europe (86.7%). Their analysis showed that sleep patterns changed significantly as they progressed from adolescence to adulthood^88^. These studies showed that women tended to sleep longer^85,86,88^^.^ Willoughby et al. examined the sleep of 226,187 Oura Ring users (54, 769, 523 nights), focusing on regional differences, and reported analyses of sleep duration, time of falling asleep, sleep quality, differences between weekdays and holidays, and studies of social jetlag^89^. A large-scale dataset collected nationwide (or globally) would facilitate a deeper understanding of the diversity and commonalities in sleep patterns, considering various factors such as seasonality, regional differences, age, educational background, and work environment. This approach could help identify optimal sleep (healthy sleep) for many individuals, thereby contributing to enhancements in sleep health.

## Conclusion

This perspective covered the concept of “sleep health,” which addresses sleep-related health comprehensively, and discussed the multidimensional evaluation of sleep. It then described wearable devices that allow for quantitative and large-scale measurement of sleep over multiple days. Following this, the concept of “sleep checkups” was explained, which involves measuring sleep with wearable devices under expert guidance and providing feedback on the results. Examples of multidimensional classification of sleep data were presented. Finally, this article addressed the potential outcomes of sleep checkups, including the accumulation of large-scale sleep data, the elucidation of sleep-health relationships through correlations with other biomarkers, and the understanding of sleep diversity.

## Data Availability

No datasets were generated or analyzed during the current study.
